# Calcium homeostasis imbalance in sepsis: molecular mechanisms and therapeutic perspectives

**DOI:** 10.3389/fmed.2026.1910574

**Published:** 2026-09-04

**Authors:** Rong Zhong, Ziyan Chen, Jie Gu, Juan Zhu, Jing Xu, Peng Jiang

**Affiliations:** 1 Department of Anesthesiology, Affiliated Hospital of Jiangsu University, Zhenjiang, Jiangsu, China; 2 Biochemistry and Molecular Laboratory, School of Life Sciences, Jiangsu University, Zhenjiang, Jiangsu, China

**Keywords:** Ca^2+^, calcium homeostasis, calcium signaling, cell death, sepsis

## Abstract

Calcium ions (Ca^2+^) are pleiotropic signaling messengers that regulate diverse cellular processes, and their homeostasis is essential for normal physiology at cellular, tissue, and organismal levels. Accumulating evidence identifies Ca^2+^ dysregulation as a key driver of sepsis onset and progression. In this review, we systematically delineate the mechanisms governing intracellular Ca^2+^ homeostasis, focusing on coordinated regulation among the endoplasmic reticulum, mitochondria, and lysosomes. We also discuss the molecular basis of disordered Ca^2+^ signaling in sepsis-associated pathologies and summarize recent therapeutic strategies targeting Ca^2+^ homeostasis, together with their translational prospects and challenges. Unlike prior reviews focusing on single organelles or individual organs, this review provides an integrative framework for organellar Ca^2+^ communication in sepsis. We propose that inflammatory insults progressively disrupt coordinated inter-organellar Ca^2+^ exchange, switching physiological coupling toward pathological states. Such aberrant inter-organellar crosstalk initiates a self-amplifying vicious cycle that ultimately propagates cellular injury to systemic multi-organ failure. Collectively, further exploration of dynamic organellar interplay within the Ca^2+^ regulatory network may yield new mechanistic insights and facilitate precise therapeutic strategies for sepsis.

## Introduction

According to the latest international definition of sepsis, sepsis is defined as life-threatening organ dysfunction caused by a dysregulated host response (1) to infection (1, 2). Numerous data indicate that sepsis represents a major global health problem, imposing a substantial burden on healthcare systems and national economies, and severely hindering sustainable human development and social progress (3, 4). With in-depth research into the mechanisms of systemic, organ, tissue and cellular dysfunction during sepsis pathogenesis, substantial advances have been made in its clinical diagnosis and treatment in recent years. Standard interventions including targeted etiological management, rational use of antimicrobials, adequate fluid resuscitation and organ supportive therapies have reduced the early-phase mortality of septic patients (1–4). Nevertheless, the overall incidence and mortality remain unacceptably high (1). A Brazilian multicenter study reported that the incidence of sepsis in intensive care units reached as high as 33%, with an associated in-hospital mortality of 55.7% (5). The latest Global Burden of Disease study confirmed that although the age-standardized incidence and mortality of sepsis have declined to different extents since 1990, an estimated 48.9 million people worldwide suffered from sepsis in 2017, resulting in 11.0 million deaths. Thus, sepsis remains one of the leading causes of health loss globally (6). Current evidence suggests that the actual disease burden of sepsis may be even more severe than previously recognized. The pathogenesis and pathophysiology of sepsis are extremely complex, involving multiple mechanisms such as genetic polymorphisms (7, 8), inflammatory network activation (9, 10), immune dysregulation (11, 12), and disorders of coagulation (13). Notably, most septic patients develop marked dysregulation of Ca^2+^ homeostasis during disease progression. This is not an isolated event, but is closely linked to the intricate pathophysiological processes of sepsis (14–16). Clinical studies have confirmed that the severity of Ca^2+^ homeostasis imbalance is strongly correlated with poor prognosis and increased mortality in septic patients (14). Therefore, elucidating the molecular mechanisms underlying dysregulated Ca^2+^ homeostasis and cellular dysfunction in sepsis, as well as exploring key therapeutic targets, is of great scientific value and clinical significance for optimizing treatment strategies and improving patient outcomes.

**Figure 1:**
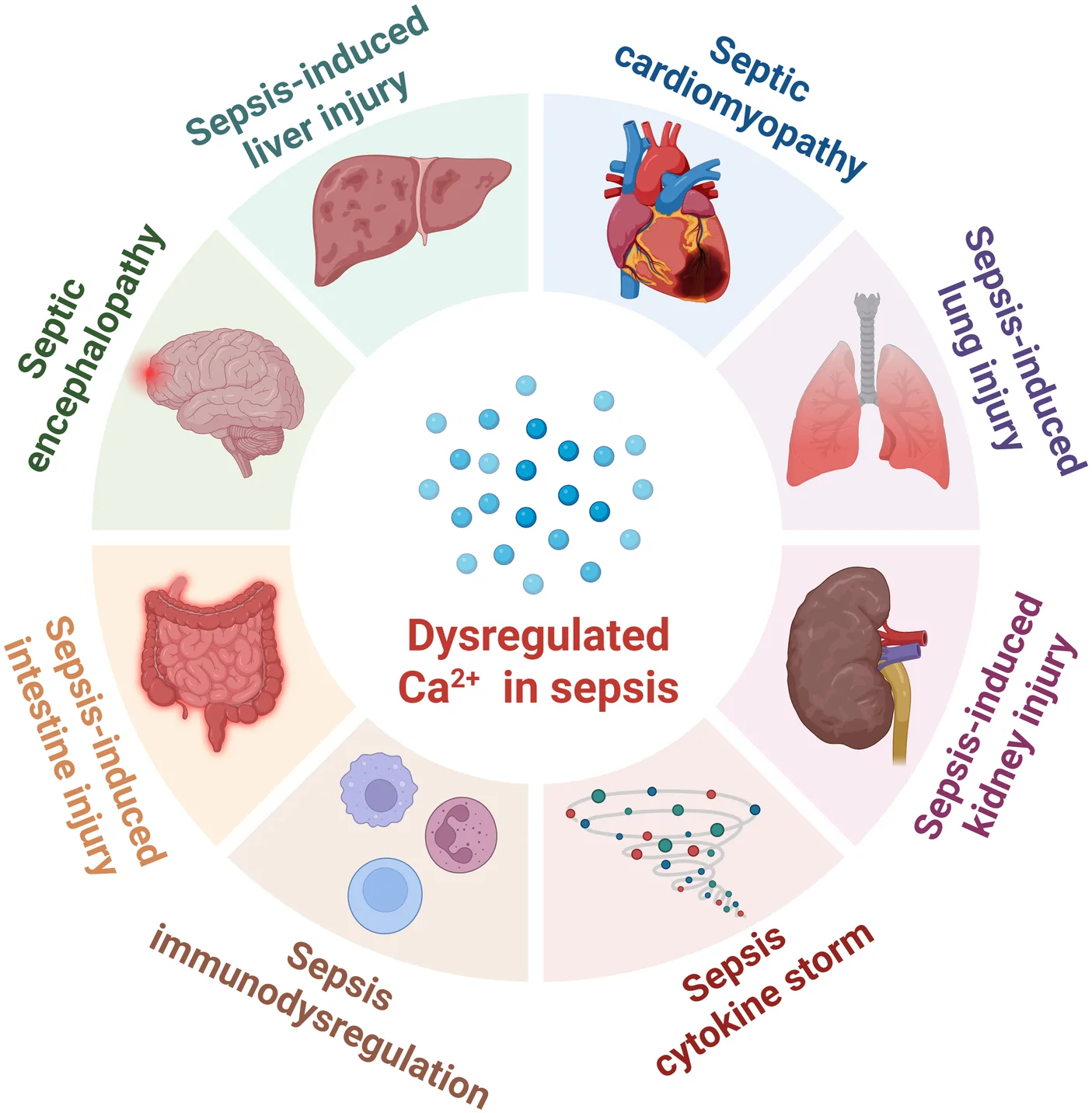
Schematic diagram illustrating the central role of dysregulated Ca^2+^ signaling in sepsis. This schematic illustrates that dysregulated Ca^2+^ homeostasis acts as an upstream driver, but not the sole pathogenic factor, of multiple sepsis-associated pathologies through multiple pathways including direct multi-organ injury, cytokine storm, and immunodysregulation. These pathways are mutually reinforcing and form a self-perpetuating vicious cycle. Normal cytosolic Ca^2+^ concentration is approximately 100 nM, whereas septic cells often exhibit sustained elevation to 500–1000 nM. Detailed molecular mechanisms for each organ system are presented in Figures 2, 3.

Ca^2+^ is a critical intracellular second messenger that widely participates in various physiological processes (17, 18). The maintenance of intracellular Ca^2+^ homeostasis depends on the precise and coordinated regulation among organelles such as the endoplasmic reticulum (ER), mitochondria, and lysosomes. Although abnormalities in Ca^2+^ signaling in sepsis have long been reported, mechanistic studies have not received sufficient attention for many years. Accumulating evidence indicates that dysregulation at any step of the above regulatory network can lead to Ca^2+^ homeostasis imbalance, thereby driving diverse sepsis-related pathological conditions (Figure 1). Accordingly, this review systematically summarizes recent advances in the Ca^2+^ regulatory network in sepsis and dissects its underlying molecular mechanisms, aiming to provide mechanistic insights and inform the development of targeted strategies for precise prevention and treatment of sepsis. Distinct from previous reviews focusing on Ca^2+^ signaling in sepsis, this work analyzes inter-organellar Ca^2+^ communication among the endoplasmic reticulum, mitochondria, and lysosomes under septic conditions, and puts forward the unifying conceptual hypothesis of “organellar Ca^2+^ coupling decoupling” to interpret sepsis-provoked cellular injury. It further highlights key unresolved knowledge gaps, including cell-type-specific Ca^2+^ signaling, spatiotemporal Ca^2+^ dynamics, and translational barriers limiting Ca^2+^-targeted therapeutics, and proposes testable hypotheses to direct future research.

## Regulation of intracellular Ca^2+^ homeostasis

As a ubiquitous second messenger, Ca^2+^ plays a central regulatory role in various life activities (17, 18). The unique divalent cation property of Ca^2+^ enables specific interactions with negatively charged macromolecules in the body, such as proteins and lipids (17, 18). Notably, during evolution from prokaryotes to higher eukaryotes, all organisms have evolved strict Ca^2+^ regulatory systems to keep cytosolic Ca^2+^ concentrations at an extremely low level (∼100 nM). This tight regulation is critical for cellular homeostasis, because excessive elevation of cytosolic Ca^2+^ can activate cell death pathways (19, 20). To maintain this delicate balance, cells have evolved a complex Ca^2+^ regulatory system, including various transporters such as Ca^2+^ pumps and Ca^2+^ channels, which are distributed on the plasma membrane and organelle membranes. By controlling Ca^2+^ influx and efflux, these proteins preserve the dynamic balance of cytosolic Ca^2+^ (19, 20). For example, store-operated Ca^2+^ entry (SOCE) is a major mechanism regulating intracellular Ca^2+^. Its key mediators are stromal interaction molecule 1 (STIM1) and the calcium release-activated calcium modulator 1 (Orai1). STIM1 serves as a Ca^2+^ sensor on the ER membrane. When ER Ca^2+^ stores are depleted, STIM1 translocates to the plasma membrane and forms highly Ca^2+^-selective pores with Orai1, mediating extracellular Ca^2+^ influx to replenish the ER Ca^2+^ stores (21). In addition, inositol 1,4,5-trisphosphate receptors (IP_3_Rs) and ryanodine receptors (RyRs) on the ER membrane mediate Ca^2+^ release from the ER into the cytosol, while sarcoplasmic reticulum Ca^2+^ ATPase (SERCA) pumps take up cytosolic Ca^2+^ into the ER to refill the internal stores. When excessive Ca^2+^ accumulates in the ER, the transmembrane and coiled-coil domain 1 (TMCO1) channel triggers store overload-induced Ca^2+^ release (SOICR), which protects cells from injury induced by Ca^2+^ overload (22). These sophisticated regulatory mechanisms together form an intricate intracellular Ca^2+^ signaling network, enabling accurate signal transduction under physiological conditions.

## Organelles in the regulation of Ca^2+^ homeostasis

The ER serves as the central hub for protein and lipid synthesis. Another core function of the ER is acting as the largest intracellular Ca^2+^ store, with luminal Ca^2+^ concentrations of 0.3–0.5 mM, three orders of magnitude above cytosolic levels (18, 19). The high Ca^2+^ level within the ER not only creates an optimal environment for ER-resident enzymes such as chaperone proteins but also enables the ER to control Ca^2+^ storage and release via channels including inositol 1,4,5-trisphosphate receptors (IP_3_Rs), thereby dynamically regulating cytosolic Ca^2+^ levels and generating specific calcium signals (19). IP_3_R has three isoforms (IP_3_R1-3) with distinct tissue distributions and differential sensitivities to oxidative modification and phosphorylation (23–26). ROS can oxidize critical cysteine residues on IP_3_R, increasing its open probability even at basal IP_3_ concentrations (27). Concurrently, inflammatory cytokines activate protein kinase C (PKC), which phosphorylates IP_3_R and further lowers its activation threshold. RyR2, the predominant isoform in cardiomyocytes, is dually regulated by redox status and PKA phosphorylation (28, 29). ROS also can oxidize RyR2 sulfhydryl groups, while PKA phosphorylation of RyR2 promotes dissociation of the stabilizing subunit FKBP12.6 (30, 31). Both modifications converge to cause diastolic SR Ca^2+^ leakage and SR store depletion. Under physiological conditions, cells maintain a delicate Ca^2+^ balance between the cytosol and organelles, sustaining sufficient Ca^2+^ storage and cytosolic Ca^2+^ buffering. Upon moderate stimulation, ER-derived Ca^2+^ release can activate cellular protective signaling pathways (19). However, under persistent pathological insults such as ER stress, excessive Ca^2+^ leaks from the ER and accumulates excessively in mitochondria and other organelles, eventually activating Ca^2+^-dependent cell death pathways (20). Besides the ER, mitochondria are also critical for the fine-tuning of intracellular Ca^2+^ homeostasis (32–34). When cytosolic Ca^2+^ rises abnormally due to extracellular Ca^2+^ influx or ER Ca^2+^ release, mitochondria take up Ca^2+^ primarily through mitochondria-associated ER membranes (MAMs) and the mitochondrial calcium uniporter (MCU), acting as an effective calcium buffer. MAMs are physical contacts between the ER and outer mitochondrial membrane, maintained by multiple tethering complexes including the IP_3_Rs-voltage-dependent anion channel (VDAC)-glucose-regulated protein 75 (GRP75) complex, mitofusin 2 (MFN2), and protein tyrosine phosphatase interacting protein 51 (PTPIP51) (35, 36). These contacts maintain a gap of approximately 10–25 nm, a distance permissive for efficient Ca^2+^, lipid, and metabolite transfer while preventing uncontrolled ion exchange (37). Upon IP_3_R-mediated Ca^2+^ release, the Ca^2+^ flux through MAMs generates a high-Ca^2+^ microdomain that is sensed by mitochondrial MCU. Ca^2+^ is subsequently transported into the mitochondrial matrix by MCU (32–34). Notably, MCU is not a single channel but a macromolecular complex comprising the pore-forming subunit MCU, the dominant-negative regulatory subunit MCUb, and regulatory subunits essential MCU regulator (EMRE), mitochondrial calcium uptake 1 (MICU1), and MICU2 (38–40). In resting cells, MICU1 and MICU2 sense Ca^2+^ in the intermembrane space and suppress MCU channel opening at low Ca^2+^ concentrations to prevent basal Ca^2+^ overload (40, 41). When ER releases Ca^2+^ via IP_3_R at MAMs, the local Ca^2+^ concentration in the microdomain can reach micromolar levels, far exceeding bulk cytosolic Ca^2+^, thereby overcoming MICU1/MICU2-mediated inhibition and activating MCU to allow rapid mitochondrial Ca^2+^ uptake (42, 43). Lysosomes have traditionally been considered degradative organelles responsible for the intracellular breakdown of biomolecules. Emerging evidence demonstrates that lysosomes also serve as critical intracellular Ca^2+^ stores and cooperate with the ER to constitute a core regulatory hub for cellular Ca^2+^ homeostasis (44, 45). ER-lysosome membrane contact sites (ER-Ly MCSs) represent specialized structural regions where the two organelles are physically tethered with a gap ranging from 10 to 30 nm, thereby enabling efficient and rapid inter-organellar Ca^2+^ exchange (45). At these contact sites, lysosomal two-pore channels (TPCs) functionally couple with ER IP_3_Rs (46). Nicotinic acid adenine dinucleotide phosphate (NAADP) activates lysosomal TPC1 to trigger local Ca^2+^ release, which generates high-Ca^2+^ microdomains that sensitize adjacent ER-localized IP_3_R1 (47, 48). This process further promotes ER Ca^2+^ efflux and establishes a lysosome-initiated Ca^2+^ signaling cascade that contributes substantially to inflammasome activation (49). In contrast, ER-to-lysosome Ca^2+^ refilling sustains lysosomal Ca^2+^ reservoir stability. This physiological process depends on intact ER-Ly MCS structure and sufficient ER Ca^2+^ reserve (47, 48). This multi-organelle Ca^2+^ regulatory network moves beyond the traditional view that emphasizes a single organelle or individual pathway, providing a systemic perspective to understand intracellular Ca^2+^ homeostasis (20). Notably, inter-organelle Ca^2+^ communication is not static but highly dynamic in both space and time. As the signaling hub, the ER precisely decodes the frequency, amplitude, and duration of Ca^2+^ release to determine whether a cell undergoes adaptation, repair, or death (20). Meanwhile, mitochondria and lysosomes are not merely passive Ca^2+^ buffers. Instead, they reciprocally modulate ER Ca^2+^ storage and release thresholds via alterations in their metabolism and membrane potential, establishing a bidirectional regulatory circuit. This inter-organelle crosstalk maintains Ca^2+^ balance under physiological conditions (32–34, 44, 45).

## Organellar crosstalk underlying inter-organellar Ca^2+^ communication in sepsis

In sepsis, pathological inflammatory stimuli profoundly remodel physical and functional contacts among organelles, thereby disrupting the finely tuned inter-organellar Ca^2+^ communication that sustains physiological homeostasis. The ER, mitochondria, and lysosomes constitute an interactive signaling network, in which Ca^2+^ transfer at membrane contact sites determines cell fate. Under septic conditions, excessive ER Ca^2+^ release mediated by inositol IP_3_Rs or RyRs leads to cytosolic Ca^2+^ overload (50, 51). Calcium is subsequently taken up by mitochondria via MAMs and MCU (50, 51). This process is particularly prominent in cardiomyocytes, where Ca^2+^/calmodulin-dependent protein kinase II (CaMKII)-dependent RyR2 phosphorylation promotes sarcoplasmic reticulum-to-mitochondria Ca^2+^ transfer, triggering mitochondrial Ca^2+^ overload and cardiomyocyte death (50). Moreover, interleukin-6 (IL-6)/signal transducer and activator of transcription 3 signaling upregulates FUN14 domain-containing 1 (FUNDC1), which enhances MAM formation and accelerates ER-to-mitochondria Ca^2+^ flux to further aggravate myocardial injury in septic mice (51). Conversely, transmembrane BAX inhibitor motif-containing 6 (TMBIM6) confers protection against septic myocardial damage by binding VDAC1, suppressing its oligomerization and diminishing Ca^2+^ translocation across the mitochondrial outer membrane (52). In the brain, sepsis-associated encephalopathy (SAE) is tightly linked to ER-mitochondria Ca^2+^ crosstalk via the IRE1α/XBP1s axis. Reduced microglial ErbB2 interacting protein (Erbin) expression induces ER stress, facilitates ER Ca^2+^ efflux, and activates the NOD-like receptor family pyrin domain containing 3 (NLRP3) inflammasome, which drives pyroptosis and hippocampal neuronal damage (53). Beyond the ER-mitochondria axis, lysosomes also contribute to Ca^2+^ homeostasis through ER-lysosome contact sites. Local rises in cytosolic Ca^2+^ sensitize IP_3_Rs and promote ER-to-lysosome Ca^2+^ uptake to alleviate ER Ca^2+^ overload (45). In sepsis, however, this lysosomal Ca^2+^-buffering capacity can be overwhelmed, perpetuating ER stress and cellular injury. In lymphoid tissues from septic mice and human patient samples, remodeling of the MCU complex perturbs mitochondrial Ca^2+^ uptake, reshapes oxidative metabolism, and heightens cellular susceptibility to apoptosis and pyroptosis, consequently amplifying systemic inflammatory responses (54). These findings support an integrative model in which sepsis disconnects physiological Ca^2+^ coupling among the ER, mitochondria, and lysosomes through three interconnected levels of dysregulation. First, excessive ER Ca^2+^ release, driven by ER stress and oxidative injury, depletes luminal Ca^2+^ reserves and compromises protein folding as well as lysosomal Ca^2+^ refilling. Second, pathological remodeling of MAMs shifts their function from signaling hubs to routes for detrimental Ca^2+^ transfer, fostering mitochondrial Ca^2+^ overload, ROS generation, and organellar dysfunction. Third, disruption of ER-lysosome contact sites impairs lysosomal Ca^2+^ buffering and autophagic clearance, weakening immune defense and cellular repair. These derangements reinforce one another in a self-perpetuating cycle: mitochondrial ROS further sensitize ER Ca^2+^ channels, lysosomal insufficiency hinders removal of damaged mitochondria, and persistent ER stress sustains the loop. Consequently, effective restoration of Ca^2+^ homeostasis may require simultaneous targeting of multiple organelles rather than a single ion channel to halt this cascade and mitigate sepsis-induced multi-organ failure. Future studies should focus on global alterations in the inter-organellar Ca^2+^ communication network and its key nodal molecules. Furthermore, exploring combination therapies targeting the restoration of multi-organelle Ca^2+^ homeostasis will open new avenues for the precise treatment of sepsis. Taken together, this three-tiered disruption of organellar Ca^2+^ communication provides a mechanistic framework to understand heterogeneous cellular and organ-specific injuries observed in clinical sepsis.

## Impaired Ca^2+^ homeostasis and its impact on sepsis

In recent years, a large number of studies have shown that Ca^2+^ homeostasis imbalance plays a critical role in the initiation and progression of sepsis and is closely associated with various sepsis-related pathological processes (Figure 1). Therefore, targeting Ca^2+^ regulation may represent a promising therapeutic strategy for sepsis. Here, we will explore the pathological role and molecular mechanisms of Ca^2+^ homeostasis imbalance in sepsis, aiming to provide new theoretical foundations and intervention targets for the clinical management of sepsis.

### Sepsis-associated encephalopathy

SAE is a frequent neurological complication affecting over half of all septic patients. However, its pathogenic mechanisms remain poorly understood (55). Recent studies using the cecal ligation and puncture (CLP) mouse model of sepsis have revealed that aquaporin-4 (AQP4) in brain astrocytes forms a complex with voltage-gated sodium channel subtype 1.6 (Nav1.6), which accelerates Na^+^ influx. The resultant rise in intracellular Na^+^ then activates the Na^+^/Ca^2+^ exchanger (NCX) and facilitates Na^+^/Ca^2+^ exchange, leading to elevated intracellular Ca^2+^ levels. Excessive intracellular Ca^2+^ overload further suppresses the peroxisome proliferator-activated receptor γ (PPAR-γ)/mechanistic target of rapamycin (mTOR) signaling axis, inhibits astrocyte autophagy and induces inflammatory responses, and eventually causes neuronal injury (56). Astrocytes and microglia can promote glutamate release, which in turn activates Ca^2+^ channels on the surface of adjacent neurons and triggers abnormally elevated intraneuronal Ca^2+^ levels. Meanwhile, excessive Ca^2+^ accumulation inside neurons damages mitochondria, induces widespread neuronal apoptosis, and aggravates brain injury in septic mice (57). Jing et al. reported that reduced expression of Erbin in microglia activates the ER stress pathway mediated by inositol-requiring enzyme 1α (IRE1α) and X-box binding protein 1 (XBP1). This process promotes ER Ca^2+^ efflux and subsequently activates the NLRP3 inflammasome. The activated NLRP3 inflammasome drives pyroptosis via the caspase-1 (CASP1)/gasdermin D (GSDMD) axis and stimulates robust release of proinflammatory cytokines, resulting in irreversible damage to hippocampal neurons and synaptic structures (53).

**Figure 2:**
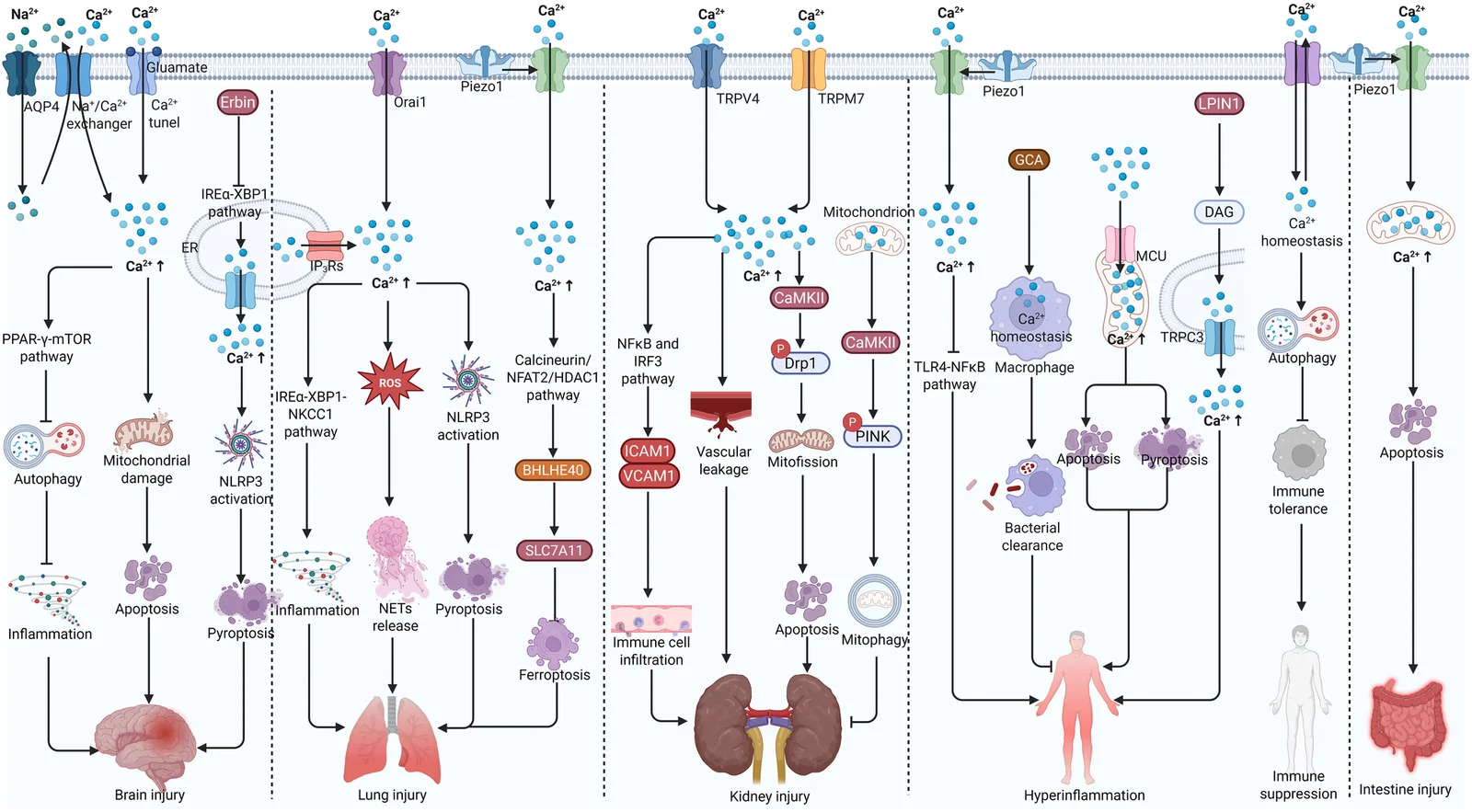
The molecular mechanisms of Ca^2+^ dysregulation underlying multiple pathological processes in sepsis. AQP4/Nav1.6 drives Na^+^ influx, NCX activation, and Ca^2+^ rise, suppressing PPARγ/mTOR and autophagy, causing inflammation and neuronal injury. Glutamate-triggered Ca^2+^ rise damages mitochondria and induces apoptosis. Reduced Erbin triggers IRE1α/XBP1 ER stress, ER Ca^2+^ efflux, NLRP3 activation, and CASP1/GSDMD pyroptosis. Orai1 Ca^2+^ influx elevates ROS and NET release. IP_3_Rs promote ER Ca^2+^ efflux and activate WNK/SPAK/NKCC1 signaling and inflammation. Piezo1 Ca^2+^ influx upregulates BHLHE40 via calcineurin/NFAT2/HDAC1 to increase SLC7A11, restraining ferroptosis. Ca^2+^ promotes NLRP3 activation, accelerating pyroptosis. TRPV4/TRPM7 Ca^2+^ influx activates NF-κB/IRF3, upregulates adhesion molecules, and leads to renal injury. Ca^2+^-activated CaMKII phosphorylates Drp1 to drive mitochondrial fission and apoptosis, but also phosphorylates PINK1 to trigger mitophagy and alleviate injury. Piezo1 Ca^2+^ influx inhibits TLR4-NF-κB to attenuate inflammation. LPIN1-derived DAG activates TRPC3, evoking ER Ca^2+^ efflux, NF-κB, and inflammation. GCA restores Ca^2+^ signaling, enhances phagocytosis, and suppresses hyperinflammation. MCU remodeling impairs mitochondrial Ca^2+^ uptake, raises mitochondrial Ca^2+^, alters metabolism, and promotes apoptosis and pyroptosis. Ca^2+^ modulation activates autophagy and alleviates immune tolerance. Piezo1 Ca^2+^ influx disrupts Ca^2+^ homeostasis and induces apoptosis, worsening injury.

Collectively, these findings demonstrate that dysregulation of Ca^2+^ homeostasis is a key mechanism underlying SAE pathogenesis, and targeting intracellular Ca^2+^ signaling represents a promising therapeutic strategy for SAE (Figure 2). Nevertheless, the aforementioned studies have several limitations. Beyond apoptosis and pyroptosis, the roles of other regulated cell death modalities, such as necroptosis and ferroptosis, in SAE remain poorly defined. It remains scarcely investigated whether these cell-death programs are activated or suppressed by Ca^2+^ signaling (58). Notably, emerging evidence suggests a bidirectional crosstalk between Ca^2+^ signaling and ferroptosis: on one hand, Ca^2+^ overload promotes ferroptosis through mitochondrial dysfunction; on the other hand, lipid peroxidation products generated during ferroptosis can reciprocally modulate Ca^2+^ channel activity (59). Furthermore, MLKL pore formation during necroptosis elevates intracellular Ca^2+^ levels, activates calpain and phospholipases, and subsequently promotes lipid peroxidation, implicating a Ca^2+^-driven “death amplification loop” between necroptosis and ferroptosis (60, 61). Collectively, these findings indicate that Ca^2+^ signaling may serve as a central hub in the cross-regulatory network of distinct cell death pathways in SAE, warranting further in-depth exploration. In addition, current research mainly focuses on acute brain injury, while the mechanisms responsible for long-term cognitive impairment during SAE progression are largely overlooked.

### Septic cardiomyopathy

**Figure 3:**
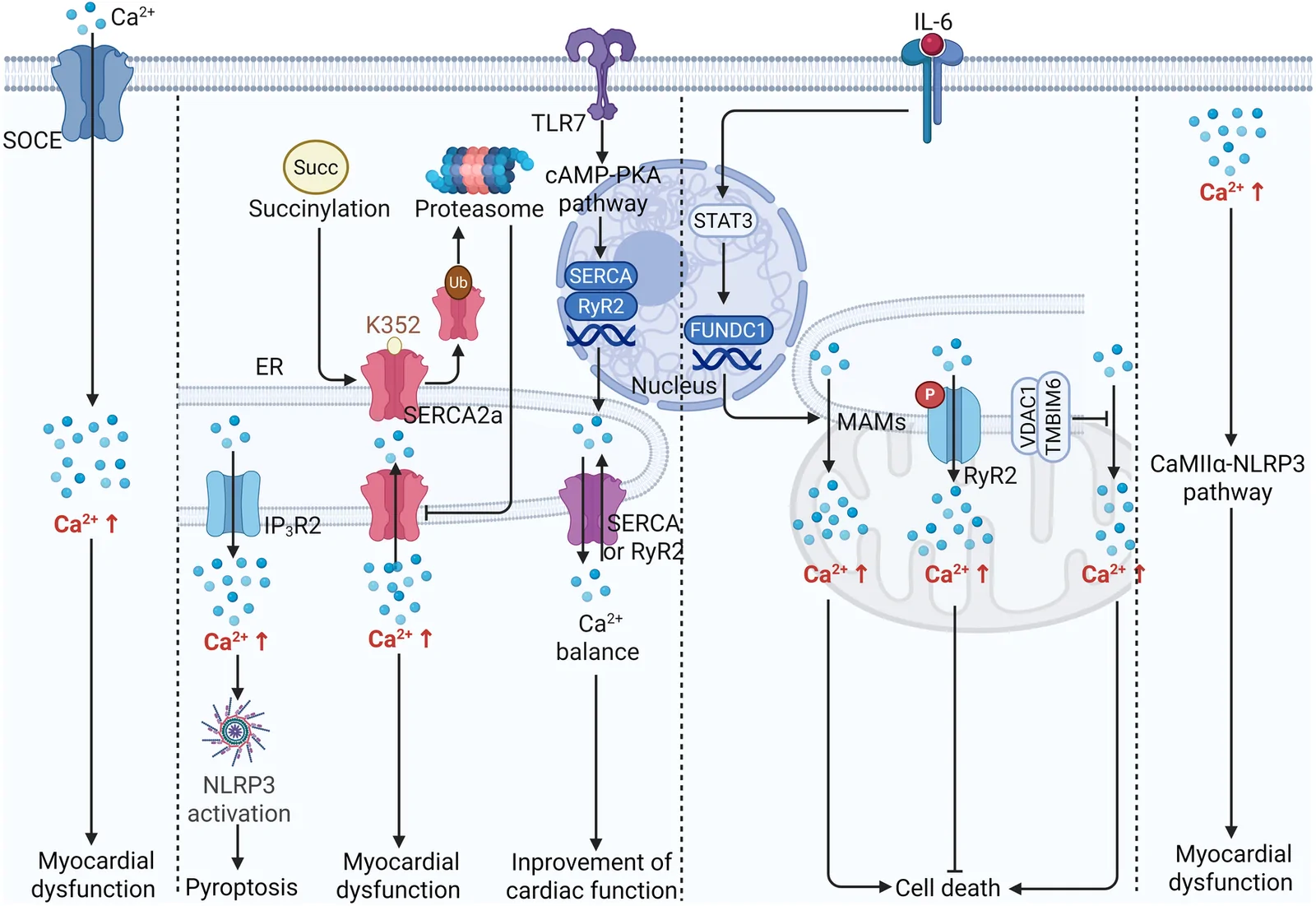
The molecular mechanism of Ca^2+^ dysregulation in the development of septic cardiomyopathy. Aberrant SOCE causes Ca^2+^ overload and myocardial depression. IP_3_R2 promotes ER Ca^2+^ release, activating NLRP3 and pyroptosis. SERCA2a succinylation at K352 triggers its ubiquitin-proteasomal degradation, causing cytosolic Ca^2+^ overload, NLRP3 inflammasome activation, and cardiomyocyte pyroptosis. TLR7 activates the cAMP-PKA pathway to upregulate SERCA and RyR2 to restore Ca^2+^ balance and improve cardiac function. IL-6/STAT3 induces FUNDC1, enhancing MAMs and ER-to-mitochondria Ca^2+^ flux via RyR2, leading to cell death. TMBIM6 binds VDAC1 to limit mitochondrial Ca^2+^ uptake, protecting cardiomyocytes. Excess Ca^2+^ activates CaMKIIα-NLRP3, promoting pyroptosis and mitochondrial oxidative stress.

Sepsis-induced cardiomyopathy (SIC) is a reversible cardiac dysfunction complicating sepsis. Patients with sepsis who develop myocardial dysfunction have markedly higher mortality. Therefore, clarifying the pathophysiological mechanisms of sepsis-related myocardial injury is critical for preventing SIC and improving clinical outcomes in septic patients. Accumulating data indicate that dysregulation of Ca^2+^ homeostasis participates in the whole progression of SIC (Figure 3). First, the Ca^2+^ channel proteins on the surface of the cardiomyocyte membrane are closely associated with intracellular Ca^2+^ homeostasis. Studies have demonstrated that aberrant activation of SOCE during sepsis causes a marked rise in intracellular Ca^2+^, which induces myocardial depression and aggravates cardiac injury in CLP-induced septic mice (62). Second, abnormal activation of Ca^2+^ flux in the sarcoplasmic reticulum can lead to intracellular Ca^2+^ homeostasis imbalance. Wu et al. reported that upregulated expression of inositol IP_3_R2 on the sarcoplasmic reticulum is closely linked to cardiomyocyte pyroptosis in septic rats (63). Mechanistically, IP_3_R2 facilitates ER Ca^2+^ efflux, which further activates the NLRP3 inflammasome and exacerbates cardiomyocyte pyroptosis and cardiac dysfunction (63). Inhibiting the expression and phosphorylation of phospholamban (PLN) curbs excessive ER Ca^2+^ release, thereby preserving cytosolic Ca^2+^ homeostasis in cardiomyocytes and protecting cardiac function during sepsis (64). Activation of Toll-like receptor 7 (TLR7) upregulates sarcoplasmic reticulum Ca^2+^ regulatory proteins, including SERCA and ryanodine receptor 2 (RyR2), via the cyclic adenosine monophosphate (cAMP)/protein kinase A (PKA) signaling pathway. This mechanism sustains Ca^2+^ homeostasis and ameliorates myocardial function in septic mice (65). In the septic state, succinylation of SERCA2a at lysine K352 promotes its K48-linked polyubiquitination and subsequent proteasomal degradation. The resultant reduction in SERCA2a expression and activity delays cytosolic Ca^2+^ clearance and lowers Ca^2+^ storage in the sarcoplasmic reticulum, ultimately impairing cardiac systolic and diastolic function (66). Third, disrupted Ca^2+^ transport across the sarcoplasmic reticulum disturbs mitochondrial Ca^2+^ homeostasis. In cardiomyocytes treated with lipopolysaccharide (LPS) to mimic sepsis, activated CaMKII phosphorylates RyR2 and promotes Ca^2+^ transfer from the sarcoplasmic reticulum to mitochondria. This triggers mitochondrial Ca^2+^ overload and accelerates cardiomyocyte death (50). The IL-6/STAT3 signaling pathway can upregulate FUNDC1, which enhances the formation of MAMs and accelerates Ca^2+^ flow from the ER into mitochondria. These changes induce cardiomyocyte death and worsen myocardial damage in septic mice (51). TMBIM6 binds to VDAC1 to suppress its oligomerization and activation, reducing Ca^2+^ transport across the outer mitochondrial membrane into the matrix. This protects mitochondria and inhibits cardiomyocyte death in septic mice (52). In addition, sepsis-triggered inflammation leads to intracellular Ca^2+^ overload in cardiomyocytes. Excess Ca^2+^ activates the CaMKIIα/NLRP3 axis, induces cardiomyocyte pyroptosis, and causes mitochondrial dysfunction and oxidative stress (67).

In summary, dysregulation of Ca^2+^ homeostasis undergoes dynamic changes during septic myocardial injury. At the early stage, inflammatory stimuli promote extracellular Ca^2+^ influx. Cells then activate compensatory mechanisms to restore homeostasis in the intermediate stage. Once Ca^2+^ overload exceeds the cellular regulatory capacity, mitochondrial dysfunction and NLRP3 inflammasome activation occur, leading to irreversible cardiac damage. This cascade of events, evolving from quantitative alterations to qualitative injury, suggests that stage-specific Ca^2+^ modulation may serve as a promising therapeutic strategy for SIC. Several caveats should be considered when interpreting findings of Ca^2+^ dysregulation in SIC. First, evidence supporting RyR2 phosphorylation and SR Ca^2+^ leakage is mainly derived from LPS-treated isolated cardiomyocytes or short-term CLP mouse models, which poorly recapitulate subacute or chronic phases of human sepsis. Second, certain molecules exert opposing biological effects; for example, CaMKII can trigger cardiomyocyte apoptosis while also driving protective PINK1-dependent mitophagy. Such duality may stem from variable Ca^2+^ stimulation strength and distinct subcellular localization of CaMKII. Third, conventional Ca^2+^ imaging probes are limited in sepsis studies: inflammation interferes with dye loading and fluorescence readouts, and these tools fail to detect Ca^2+^ within MAM microdomains.Collectively, these limitations emphasize that current conclusions for SIC-related Ca^2+^ channel dysfunction require further validation in more physiologically pertinent models, such as human iPSC-derived cardiomyocytes or humanized mice.

### Sepsis-induced lung injury

Sepsis is a life-threatening critical illness, and multiple organ dysfunction syndrome represents its most severe complication. The lung is the most vulnerable target organ during this pathological process (68, 69). Clinicians still lack reliable tools for early detection and accurate diagnosis of sepsis-induced lung injury in routine clinical practice. Niu et al. demonstrated that knocking down the expression of the Ca^2+^ channel Orai1 on neutrophils, or using selective inhibitors to block Orai1 activity, markedly diminished extracellular Ca^2+^ influx (70). This effect reduced intracellular reactive oxygen species (ROS) accumulation and neutrophil extracellular trap (NET) release, ultimately alleviating pulmonary inflammation in septic mice (70). Another study showed that LPS stimulation inhibits the activation of IP_3_Rs on the ER of lung epithelial cells, significantly reducing ER Ca^2+^ efflux, thereby suppressing the activation of the With No Lysine Kinase (WNK)/STE20/SPS1-related Proline/Alanine-rich Kinase (SPAK)/Na^+^-K^+^-2Cl^−^ Cotransporter 1 (NKCC1) signaling pathway and decreasing the release of inflammatory cytokines (71). During sepsis, fluid shear stress activates the endothelial mechanosensitive channel Piezo1 and triggers Ca^2+^ influx. The increased Ca^2+^ then upregulates the transcription factor helix-loop-helix transcription factor 40 (BHLHE40) through the calcineurin/nuclear factor of activated T-cells 2 (NFAT2)/HDAC1 axis. BHLHE40 subsequently elevates solute carrier family 7 member 11 (SLC7A11) expression, restrains endothelial ferroptosis, and relieves pulmonary vascular leakage and inflammation (72). Additionally, excessive intracellular Ca^2+^ in macrophages promotes the oligomerization of apoptosis-associated speck-like protein containing a CARD (ASC) and activates the NLRP3 inflammasome. This cascade accelerates pyroptosis and aggravates lung inflammation in septic mice (73). Several limitations should be acknowledged. First, most evidence derives from LPS-based models that poorly reflect polymicrobial sepsis, and findings from neutrophils or endothelial cells require verification in CLP models and human samples. Second, Piezo1 exerts opposing effects across cell types. It is protective in endothelium (72) but detrimental in intestinal epithelium (74), which complicates systemic targeting. Third, LPS-induced IP_3_R inhibition in lung epithelial cells (71) contradicts the widespread ER Ca^2+^ depletion observed elsewhere, raising questions of cell-specificity versus experimental variability. Finally, clinical validation as a biomarker is lacking, and spatiotemporal Ca^2+^ dynamics in the lung remain unexplored (Figure 3).

### Sepsis-induced kidney injury

In sepsis, the kidneys are among the earliest organs to sustain damage. Approximately 60% of patients with septic shock develop acute kidney injury (AKI), which increases mortality 6- to 8-fold (75, 76). Accumulating studies have demonstrated that dysregulation of intracellular Ca^2+^ homeostasis plays a pivotal role in the onset and progression of sepsis-associated AKI (Figure 3). Wang et al. reported that in septic mice, the expression of transient receptor potential vanilloid 4 (TRPV4) in glomerular endothelial cells was markedly upregulated, which mediated excessive extracellular Ca^2+^ influx (77). Elevated intracellular Ca^2+^ further activated the nuclear factor-κB (NF-κB)/interferon regulatory factor 3 (IRF3) inflammatory pathway, boosted the production of intercellular adhesion molecule-1 (ICAM-1) and vascular cell adhesion molecule-1 (VCAM-1), and aggravated renal injury via enhanced immune cells infiltration (77). Another study showed that transient receptor potential melastatin 7 (TRPM7) on glomerular endothelial cells also facilitated extracellular Ca^2+^ influx in septic mice, resulting in increased glomerular vascular permeability and subsequent renal dysfunction (78). Beyond glomerular endothelial cells, Ca^2+^ overload in renal tubular epithelial cells activates CaMKII in LPS-treated septic rats. Activated CaMKII phosphorylates dynamin-related protein 1 (Drp1), driving excessive mitochondrial fission and ultimately triggering cell apoptosis and renal damage (79). Notably, Zhang et al. found that disrupted mitochondrial Ca^2+^ homeostasis in renal cells from LPS- and CLP-induced septic mice also activated CaMKII (80). This kinase mediates the phosphorylation of PTEN-induced putative kinase 1 (PINK1) and further initiates mitophagy, which alleviates mitochondrial damage and mitigates kidney injury (80). These findings suggest that Ca^2+^ signals in different subcellular compartments exert divergent regulatory effects on cell fate. However, several caveats should be considered. First, most evidence derives from rodent models with limited validation in human septic AKI, and species differences constrain the translational relevance of TRPV4 and TRPM7 findings. Second, CaMKII exerts opposing effects in tubular cells, promoting apoptosis (79) while also initiating protective mitophagy (80), yet the conditions governing this balance remain undefined. Third, the causal role of Ca^2+^ dysregulation in renal injury requires rigorous establishment, as many associations may reflect epiphenomena rather than primary drivers. Further investigations are required to clarify the full molecular regulatory network underlying these processes.

### Hyperinflammation and immunosuppression in sepsis

Evidence demonstrates that sepsis is driven by a dynamic imbalance between pro-inflammatory and anti-inflammatory responses. In the early phase, pro-inflammatory reactions predominate, accompanied by severe cytokine storms and vascular endothelial damage. As the disease advances, the immune system shifts toward an anti-inflammatory phenotype, resulting in immune dysfunction and higher susceptibility to secondary infections (1, 81). Liu et al. reported that activation of the mechanosensitive ion channel Piezo1 facilitates extracellular Ca^2+^ influx in macrophages (82). The resultant elevation of intracellular Ca^2+^ attenuates inflammation in septic mice through inhibition of the TLR4-NF-κB pathway (82). Another study revealed that upon LPS challenge, lipin 1 (LPIN1) in macrophages modulates intracellular Ca^2+^ homeostasis and inflammatory signaling by regulating diacylglycerol (DAG) production (83). Specifically, LPIN1-generated DAG activates transient receptor potential canonical 3 (TRPC3) on the ER membrane, which accelerates ER Ca^2+^ efflux, boosts NF-κB pathway activity and aggravates inflammation (83). The calcium-binding protein grancalcin (GCA) restores Ca^2+^ signaling homeostasis in macrophages, enhances bacterial phagocytosis, suppresses hyperinflammation, and mitigates multiple organ injury (84). In lymphoid tissues of septic mice and human, remodeling of the mitochondrial calcium uniporter (MCU) complex disrupts mitochondrial Ca^2+^ uptake and elevates mitochondrial Ca^2+^ levels. This disturbance alters cellular oxidative metabolism, increases cell vulnerability to apoptosis and pyroptosis, and exacerbates systemic inflammation (54). Beyond mediating hyperinflammation, dysregulation of Ca^2+^ homeostasis is also tightly linked to sepsis-induced immunosuppression. Endotoxin tolerance describes a blunted inflammatory response of cells such as macrophages to repeated or persistent LPS stimulation, and it is a major driver of immunosuppression during sepsis (85). Liu et al. found that modulation of intracellular Ca^2+^ homeostasis activates autophagy and alleviates LPS tolerance in macrophages (68). This finding identifies a promising therapeutic target for treating sepsis-related immunosuppression. In T cells, functional CaSR expression enables inflammatory stimuli to trigger intracellular ROS accumulation. Importantly, T cell receptor (TCR) activation strictly relies on STIM1-Orai1-mediated SOCE, which sustains NFAT nuclear translocation and supports T cell proliferation. During sepsis, pronounced impairment of T cell SOCE disrupts this signaling axis, leading to suppressed T cell proliferation and cytokine secretion. Such Ca^2+^ signaling dysfunction is well recognized as a core mechanism driving sepsis-induced T cell anergy (15, 86). Notably, other lymphoid populations, including B cells and NK cells, are also highly vulnerable to septic Ca^2+^ dysregulation. Unlike T lymphocytes, B cells lack cell-surface CaSR expression. Nevertheless, B-cell receptor (BCR) signal transduction is also critically dependent on SOCE-mediated Ca^2+^ influx. Although Ca^2+^ signals are presumed to modulate B-cell-mediated antibody production and immunoglobulin class-switching, mechanistic studies exploring B-cell Ca^2+^ signaling under septic conditions remain very scarce, and the precise regulatory mechanisms have yet to be defined (87). In NK cells, cytotoxicity is tightly controlled by Ca^2+^-dependent granule exocytosis. Impaired Ca^2+^ signaling is speculated to partially account for sepsis-associated NK cell exhaustion and functional suppression, though direct experimental evidence remains insufficient (88, 89).

Collectively, these studies indicate that Ca^2+^ signaling exerts dual regulatory effects in sepsis: it contributes to the early hyperinflammatory cascade and also mediates the subsequent immunosuppressive state. Accordingly, targeting Ca^2+^ homeostasis allows stage-specific intervention against sepsis and provides valuable clues for developing innovative therapeutic approaches (Figure 3).

### Other sepsis-associated disorders

Zhou et al. (90) found that the *NLRP3* T346M mutation promotes the activation of the pyroptosis signaling pathway and exacerbates LPS-induced inflammatory responses and liver injury in septic mice, a process closely associated with intracellular Ca^2+^ homeostasis imbalance; however, the specific mechanisms remain to be further elucidated. Piezo1 has been shown to mediate extracellular Ca^2+^ influx in small intestinal epithelial cells, leading to intracellular and mitochondrial Ca^2+^ dysregulation, activating apoptosis, and exacerbating intestinal injury in septic mice (74) (Figure 3). A retrospective study based on the MIMIC-III database (*n* = 3,016) revealed a *U*-shaped association between total serum calcium levels and 28-day mortality in septic patients, with a threshold point of 9.0 mg/dL. When serum calcium was below 9.0 mg/dL, each 1.0 mg/dL decrease was associated with a 58% increase in mortality risk; when above 9.0 mg/dL, each 1.0 mg/dL increase was associated with a 12% increase in mortality risk. Patients in the lowest calcium quintile (<8.20 mg/dL) had a 112% higher risk of 28-day mortality compared with the reference group. This study was the first to quantify the nonlinear relationship between serum calcium and short-term prognosis in a large sepsis cohort, suggesting that serum calcium may serve as a convenient risk-stratification biomarker. However, the study measured total rather than ionized calcium and did not adjust for confounders such as fluid resuscitation; therefore, the causal relationship remains to be validated in prospective studies (14).

### Therapeutic strategies targeting Ca^2+^ homeostasis

Based on the important role of Ca^2+^ homeostasis in sepsis, drugs that regulate Ca^2+^ homeostasis show potential therapeutic prospects for the treatment of sepsis. Currently, drugs targeting Ca^2+^ homeostasis mainly include plant-derived extracts, hormonal agents, and synthetic compounds.

### Plant-derived extracts

Recent studies demonstrate that plant-derived compounds targeting Ca^2+^ homeostasis possess distinct advantages including natural origins, good safety and multi-target synergistic effects. These agents exhibit great potential for clinical translation in sepsis therapy. The extract of Descurainiae semen (tinglizi) maintains Ca^2+^ homeostasis in alveolar lavage cells via inhibiting IP_3_R activation on the endoplasmic reticulum membrane, and thus suppresses LPS-induced production and release of proinflammatory cytokines (71). Chlorogenic acid directly binds to the GLU60 site of CaMKIIα, restrains excessive activation of the Ca^2+^-CaMKIIα signaling pathway, and prevents NLRP3 inflammasome assembly and activation. This intervention reduces cardiomyocyte pyroptosis and ultimately improves cardiac function in sepsis-induced cardiomyopathy (67). Artesunate, a water-soluble derivative of artemisinin, alleviates LPS-induced endotoxin tolerance in macrophages by modulating intracellular Ca^2+^ homeostasis, offering a novel strategy to treat sepsis-related immunosuppression (68). Nevertheless, the exact molecular mechanisms underlying the regulatory effect of artesunate on intracellular Ca^2+^ homeostasis remain to be further elucidated.

### Hormonal agents

Thyroid hormones are synthesized by thyroid follicular epithelial cells and stored within the follicular lumen. Their biologically active forms are thyroxine (T₄) and triiodothyronine (T_3_) (91). Studies have demonstrated that T_3_ inhibits the expression and phosphorylation of PLN in the sarcoplasmic reticulum. This action reduces ER Ca^2+^ efflux, preserves Ca^2+^ homeostasis in cardiomyocytes and protects cardiac function in septic mice. Such protective effects are mediated via the c-Jun N-terminal kinase (JNK)/Jun proto-oncogene (c-Jun) signaling pathway (64). Dexamethasone (DEX), a potent synthetic glucocorticoid, possesses strong anti-inflammatory, anti-endotoxin and anti-allergic activities (92). Liu et al. (82) verified that DEX upregulates Piezo1 expression on macrophages and facilitates Ca^2+^ influx. This helps maintain cellular Ca^2+^ homeostasis and curbs LPS-induced release of proinflammatory cytokines. These findings suggest that hormonal agents have promising prospects for the prevention and treatment of sepsis. Nevertheless, their clinical efficacy and safety need further rigorous evaluation. Moreover, hormones including melatonin and estrogen are known to alleviate retinal degeneration and cardiovascular disorders by modulating Ca^2+^ homeostasis. Their roles and underlying mechanisms in sepsis remain largely uncharacterized (93–95).

### Synthetic compounds

Dexmedetomidine is a highly selective α_2_-adrenoceptor agonist with sedative, analgesic and sympatholytic activities (96). Unlike other sedatives, it exerts prominent organ-protective effects via its anti-inflammatory, anti-oxidative and anti-apoptotic properties (97). Recent studies using LPS-challenged septic rat models have shown that dexmedetomidine modulates intracellular Ca^2+^ homeostasis to preserve mitochondrial membrane dynamics, protect cells against oxidative injury and alleviate sepsis-associated kidney injury (79). Memantine hydrochloride is a noncompetitive N-methyl-D-aspartate receptor (NMDAR) antagonist and belongs to the adamantane derivatives. It is clinically approved primarily for moderate to severe Alzheimer's disease (98). Ding et al. demonstrated that memantine hydrochloride markedly inhibits extracellular Ca^2+^ influx in macrophages. This effect attenuates NLRP3 inflammasome activation, suppresses LPS-induced pyroptosis, and reduces the release of proinflammatory cytokines (73). Spiperone is a typical butyrophenone antipsychotic agent that treats schizophrenia by blocking cerebral dopamine and serotonin receptors. In sepsis, spiperone activates the Phospholipase C (PLC)/IP_3_R pathway to facilitate Ca^2+^ influx and trigger endothelium-dependent vasodilation. These actions improve endothelial dysfunction, mitigate systemic inflammation and confer organ protection (99). Esketamine, the dextrorotatory isomer of ketamine, possesses stronger NMDA receptor antagonism and a rapid onset of action. It is mainly used for treatment-resistant depression. In research on SAE, esketamine modulates neuronal Ca^2+^ signaling and activates the NFAT1/cAMP response element-binding protein (CREB) pathway. It alleviates neuroinflammation, restores synaptic integrity and consequently improves cognitive function (100). In addition, magnesium sulfate (MgSO₄) inhibits the NF-κB pathway, relieves mitochondrial Ca^2+^ overload in LPS-stimulated renal tubular epithelial HK-2 cells, and suppresses inflammatory responses and apoptosis, thereby exerting renoprotective effects (101). Although these drugs were initially developed for other indications, current findings have uncovered their new roles in sepsis and provided additional therapeutic options for clinical practice.

Additionally, several fundamental challenges complicate Ca^2+^-targeted therapy for sepsis. First, the narrow therapeutic window: Ca^2+^ mediates vital physiological functions, so systemic modulation risks off-target toxicity. The U-shaped relationship between serum calcium and 28-day mortality in septic patients (14) requires tight homeostatic control during intervention. Second, agents exert disease-stage-dependent effects: Ca^2+^ overload dominates early hyper-inflammation, while lymphocyte Ca^2+^ defects arise in late immunosuppression. Most pre-clinical models initiate treatment at sepsis onset and cannot mirror patients presenting at variable disease stages. Third, critical-illness-driven pharmacokinetic changes from resuscitation, capillary leakage and organ dysfunction render animal-derived dosages poorly translatable. Fourth, tissue-specific delivery remains pre-clinical despite nanoparticle and extracellular-vesicle strategies. Fifth, clinical validation is lacking: no randomized controlled trials test intracellular Ca^2+^-targeting therapeutics, and existing evidence focuses only on serum calcium adjustment. Repurposed agents are supported chiefly by rodent LPS/CLP experiments with minimal human data. Collectively, these barriers account for the absence of approved Ca^2+^-directed sepsis therapies and highlight the urgent need for high-quality clinical trials.

## Conclusion and future directions

In summary, calcium ions (Ca^2+^) act as ubiquitous second messengers, and their dysregulated homeostasis is a key driver of sepsis progression. This review summarizes coordinated Ca^2+^ regulation mediated by the endoplasmic reticulum, mitochondria, and lysosomes, and describes how aberrant Ca^2+^ signaling contributes to sepsis-associated organ injury, hyperinflammation, and immunosuppression. Multiple natural and synthetic Ca^2+^-targeted agents have shown protective effects in preclinical animal models and hold translational promise. Nevertheless, substantial obstacles remain, including incomplete understanding of multi-organelle Ca^2+^ networks, technical constraints in measuring spatiotemporal Ca^2+^ dynamics, poorly defined therapeutic windows and off-target risks—and, perhaps most critically, the translational limitations of preclinical sepsis models. LPS injection reflects only endotoxin effects and fails to capture host-pathogen dynamics, whereas CLP, though more clinically relevant, suffers from high inter-laboratory variability. More fundamentally, rodents and humans differ significantly in immune composition and Ca^2+^ signaling components, so agents effective in animals may be ineffective—or even deleterious—in humans. Furthermore, the typical 1- to 6-hour drug administration window in preclinical studies does not mirror the later-stage presentation of septic patients, a temporal mismatch that may underlie many translational failures. Critically, no high-quality randomized controlled trial has yet validated any Ca^2+^-targeted intervention in septic patients; the existing evidence is confined to retrospective analyses of serum calcium correction, which does not equate to targeted modulation of intracellular Ca^2+^ signaling. Therefore, all therapeutic strategies discussed in this review should be regarded as preclinical proof-of-concept, not as clinically applicable therapies.

Notably, cell-type-specific Ca^2+^ signaling represents one major unresolved gap. Different cell populations possess distinct Ca^2+^-handling machineries and respond heterogeneously to septic stress. Cardiomyocytes rely primarily on RyR2-mediated sarcoplasmic reticulum Ca^2+^ release; its abnormal phosphorylation induces mitochondrial Ca^2+^ overload and cell death (50). Immune cells such as macrophages and neutrophils utilize STIM1-Orai1-dependent store-operated Ca^2+^ entry to govern inflammatory activation and pyroptosis (21). Endothelial cells employ Piezo1-driven Ca^2+^ influx to control vascular permeability and ferroptosis, whereas renal parenchymal cells develop pathological Ca^2+^ overload largely via TRP-family channels, triggering mitochondrial fission and apoptosis (72, 77, 78). Given the lack of direct head-to-head comparative investigations, generalized Ca^2+^-targeted therapies may yield inconsistent or even opposing effects across cell types, highlighting the need for cell-type-tailored therapeutic strategies.

Beyond cell-type differences, many Ca^2+^-related molecules exert context-dependent, seemingly conflicting protective or detrimental functions, which stem from the pleiotropic nature of Ca^2+^ signaling rather than genuine experimental contradictions. Three key determinants govern these divergent functional outcomes. First, cell-type background: Piezo1-driven Ca^2+^ influx induces apoptosis in intestinal epithelial cells (74) but inhibits TLR4-NF-κB-mediated inflammation in macrophages (82), due to cell-specific downstream signaling circuits. Second, subcellular localization: CaMKII triggers tubular epithelial apoptosis via Drp1-driven mitochondrial fission (79), while mitochondria-associated CaMKII promotes protective PINK1-dependent mitophagy (80); such opposing activities may occur sequentially. Third, stimulus magnitude and duration: mild transient Ca^2+^ signals elicit adaptive responses including autophagy, whereas persistent excessive Ca^2+^ elevation activates cell-death pathways, as illustrated by dose-variant CaMKII function. Accordingly, individual molecules cannot be simply categorized as exclusively protective or detrimental. Their biological effects must be interpreted by integrating cell-type features, subcellular distribution, and the strength and duration of pathological stimulation.

Although advances have been made in understanding inter-organellar Ca^2+^ communication in sepsis, several critical questions merit further exploration. What molecular constituents mediate dynamic remodeling of ER-lysosome contact sites during sepsis, and what are the core underlying mechanisms? Does sepsis-triggered inflammation modify tethering-protein expression and post-translational status to diminish ER-lysosome contacts and impair lysosomal Ca^2+^ refilling? How can we precisely resolve spatiotemporal Ca^2+^ dynamics, particularly local Ca^2+^ microdomain signals at organellar contact sites? Do pathological Ca^2+^ rises within MAM microdomains precede global cytosolic Ca^2+^ overload, and can this early time-window be harnessed for intervention? What are the optimal timing and targeting principles for Ca^2+^-modulating treatments? Would Ca^2+^-channel blockade confer protection in early hyper-inflammatory sepsis yet cause harm during late immunosuppression? To address these open questions, future work should aim to construct comprehensive Ca^2+^ regulatory networks, develop high-resolution Ca^2+^ imaging tools, dissect cell-specific mechanisms, screen selective Ca^2+^ modulators combined with nanodelivery platforms, and perform clinical studies for biomarker validation and drug repurposing. Deep mechanistic insights into inter-organellar Ca^2+^ crosstalk will facilitate the development of targeted therapies and improve clinical outcomes for septic patients.
